# Enterovirus D68–Associated Acute Flaccid Myelitis in Immunocompromised Woman, Italy

**DOI:** 10.3201/eid2310.170792

**Published:** 2017-10

**Authors:** Emanuela Giombini, Martina Rueca, Walter Barberi, Anna Paola Iori, Concetta Castilletti, Paola Scognamiglio, Francesco Vairo, Giuseppe Ippolito, Maria Rosaria Capobianchi, Maria Beatrice Valli

**Affiliations:** National Institute for Infectious Diseases “L. Spallanzani”–Istituto di Ricovero e Cura a Carattere Scientifico, Rome, Italy (E. Giombini, M. Rueca, C. Castilletti, P. Scognamiglio, F. Vairo, G. Ippolito, M.R. Capobianchi, M.B. Valli);; “Sapienza” University, Rome (W. Barberi, A.P. Iori, M. Inghilleri);; Lazio Regional Service for the Epidemiology and Control of Infectious Diseases, Rome (P. Scognamiglio, F. Vairo)

**Keywords:** Enterovirus D68, human, central nervous system infection, VP1 sequencing, molecular epidemiology, immunocompromised patient, viruses, Italy, acute flaccid myelitis

## Abstract

In Italy in 2016, acute flaccid myelitis developed in a woman who had received a hematopoietic stem cell transplant. Enterovirus D68 viral genome was detected in respiratory and cerebrospinal fluid samples, and the viral protein 1 sequence clustered with lineage B3. Immunocompromised adults may be at risk for enterovirus D68–associated neurologic complications.

Enteroviruses are the most common viruses circulating worldwide and lead to a broad spectrum of clinical illnesses: respiratory infection; hand, foot, and mouth disease; acute and chronic cardiac disease; meningitis; and encephalitis (*1*). Originally isolated in 1962 from respiratory specimens of children with severe pneumonia, enterovirus serotype D68 (EV-D68), formerly classified as rhinovirus 87, belongs to the *enterovirus D* species (*2*). EV-D68 infection typically causes mild respiratory illness but occasionally may progress to more severe clinical syndromes (pneumonia, hepatitis, cardiomyopathy, and acute neurologic diseases including aseptic meningitis and poliolike paralytic disease). 

Since its initial identification, EV-D68 has been rarely identified, but more recently, it has become increasingly recognized in the context of enhanced surveillance for poliolike diseases (*3*). The extent of EV-D68 circulation is underestimated because of the scarcity of laboratories equipped to detect it and poor awareness among physicians. However, in the past decade, EV-D68 has emerged as a major respiratory pathogen, especially in children (*1**,**4*). Moreover, concurrent with the unprecedented respiratory outbreak of EV-D68 in North America in 2014, an apparent increased incidence of acute flaccid myelitis (AFM), consistent with poliolike illness, has been reported in several US states (*5*). These neurologic cases have been temporally associated with EV-D68 infection, although virus sequences were detected almost exclusively in respiratory specimens. Concurrently, EV-D68 was circulating in Europe, where the disease burden was more moderate than in the United States (*4*). Indeed, EV-D68 has been only rarely detected in the cerebrospinal fluid (CSF) of children with neurologic involvement (*6**,**7*). 

A direct causative role of the virus in neurologic disease needs further evidence. We report a fatal case of EV-D68 infection and AFM in an adult recipient of a hematopoietic stem cell transplant (HSCT).

## The Case

In October 2016, a 55-year-old woman was admitted to the emergency unit of the Hematology Department of Policlinico Umberto I (Rome, Italy) for sudden acute weakness and limited mobility of the left arm. The woman had no history of preexisting neurologic disease or recent travel, but she had had follicular non-Hodgkin B-cell lymphoma since 2011, which evolved into a diffuse large B-cell lymphoma despite immunochemotherapy and corticosteroid treatment. She underwent 2 HSCTs: 1 autologous in 2011 and 1 allogeneic in 2013. Despite treatment with cyclosporine and methotrexate, acute graft-versus-host disease with liver and gut involvement (grade IV) developed and was treated with high-dose corticosteroids and extracorporeal photopheresis. Although the graft-versus-host disease initially evolved into a severe chronic form, 1 year after allogeneic HSCT, the patient’s clinical condition gradually improved, and the immunosuppressive therapy was slowly reduced. At the 2016 hospital admission, she was receiving only a minimal dose of corticosteroids (prednisone 10 mg/d) and mycophenolate (750 mg/d).

**Figure 1:**
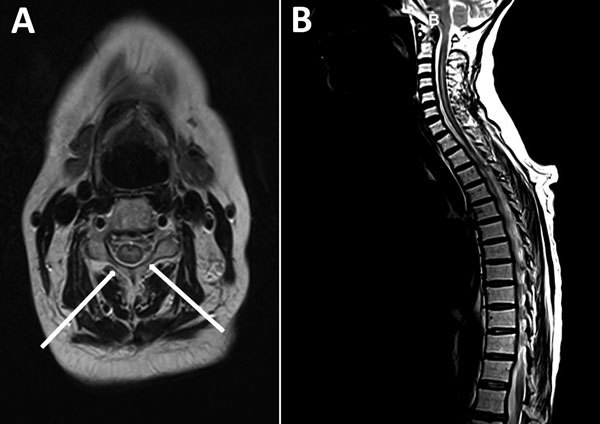
Magnetic resonance images of the brain and spinal cord of a woman who later died of fatal neurologic disease associated with enterovirus D68 infection. A) Sagittal and axial T2 image of the spinal cord showing cord swelling, particularly at the cervical level (arrows). B) Extensive hyperintensity in the central cord.

A few days before onset of neurologic illness, the patient had had mild fever without respiratory or gastrointestinal symptoms. At admission, she had no fever, and her lymphocyte count was within reference range (5.25 × 10^9^ cells/L). A few hours after admission, muscle weakness extended to the right arm, neck, and head and evolved into tetraplegia with proximal muscle involvement. No deficits of superficial sensitivity or dysphagia were recorded. Initial therapy at admission was acyclovir, trimethoprim/sulfamethoxazole, and ciprofloxacin. CSF analysis was consistent with aseptic meningitis with pleocytosis (130 leukocytes/mm^3^, 92.6% mononucleocytes), mildly elevated glucose level (86 mg/dL), and protein concentrations within reference range (34 mg/dL). Magnetic resonance images of the brain and spinal cord (Figure 1) showed a bulging spinal tract rope at C2–D1 with hyperintensity in the central region, which increased after administration of gadolinium, involving mainly the medullar anterior horn and sparing the posterior columns and lateral and ventral fins. The findings fulfilled the US Centers for Disease Control and Prevention and the California Public Health Department criteria for AFM (*8*). The images showed no signs of white matter involvement.

Electromyograms showed signs of C4–C7 horn abnormality, more evident on the left side, with absence of F wave at the upper limbs. The patient’s neurologic function rapidly worsened to include respiratory muscles. Intubation and ventilator support failed to improve respiratory function, and the patient died 14 days after symptom onset.

Clinical specimens (CSF, blood, oropharyngeal swab, and feces) collected 3 days after admission were sent to the regional reference laboratory in Rome (National Institute for Infectious Diseases “Lazzaro Spallanzani”), where the CSF was immediately analyzed by FilmArray ME (bioMérieux, Marcy l’Etoile, France), a molecular test that can rapidly detect many neurotropic pathogens; the test was positive for enteroviruses only. To confirm the enterovirus diagnosis, a panel of molecular and serologic tests was performed. Enterovirus genomes were detected by a commercial reverse transcription quantitative PCR (REALQUALITY RQ-ENTERO; AB-Analatica, Padova, Italy) in CSF and oropharyngeal swab samples (cycle thresholds 36.61 and 34.94, respectively), but not in feces, consistent with the fact that respiratory samples are the best diagnostic specimens for this enterovirus. Both samples also produced positive results in 2 laboratory-developed reverse transcription PCRs targeting the 5′ untranslated region and viral protein 1 (VP1) (*9*). Screening of CSF for the presence of genomes of other known neurotropic viruses (flaviviruses, cytomegalovirus, Epstein-Barr virus, herpes simplex viruses 1 and 2, varicella zoster virus), and serologic tests (for coxsackieviruses, adenoviruses, polioviruses) yielded negative results.

**Figure 2:**
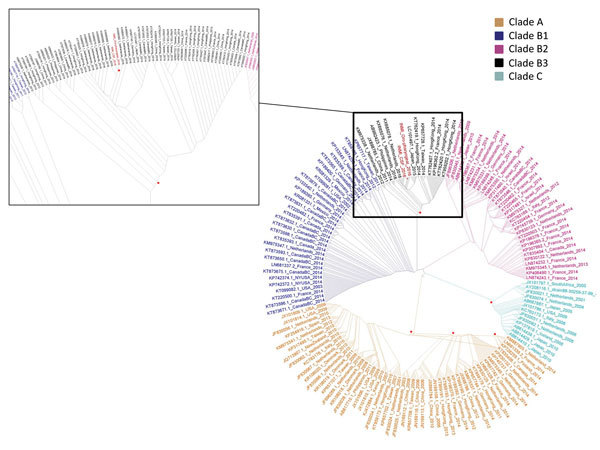
Phylogenetic tree of partial viral protein 1 sequences of 279 nt (nt positions 2581–2859, reference sequence GenBank accession no.AY426531.1), sequences from cerebrospinal fluid (accession no. MF061604) and oropharyngeal swab sample (accession no. MF061605) from a woman who died of fatal neurologic disease associated with enterovirus-D68 infection (indicated in red) in the context of 918 enterovirus-D68 global sequences retrieved from the National Center for Biotechnology Information (https://www.ncbi.nlm.nih.gov/). The main figure shows the whole maximum-likelihood tree (1,000 bootstrap replicas), generated by using the HKY+GI (Hasegawa-Kishino-Yano + gamma distribution invariant sites) model; the inset shows an enlargement of subclade B3 containing the sequences from the patient reported here and patients involved in the 2016 epidemic in the Netherlands: the closest sequence is KX685068.1_Netherlands_2016 (98% identity, distance: 180 substitutions/10^4^ positions). Red dots indicate nodes with bootstrap value >70.

The amplicons targeting the 5′ untranslated region and VP1 were sequenced by using the Sanger method. The genus/species classification of the enterovirus genotype as D68 was based on the web-based open-access Enterovirus Genotyping Tool version 0.1 (*10*). Phylogenetic analysis of VP1 was performed in the context of 918 EV-D68 worldwide sequences retrieved from GenBank. Clades were assigned according to Tokarz et al. (*3*). In the resulting maximum-likelihood tree (Figure 2), the virus involved in this case clustered with the recently described subclade B3, comprising also the viral sequences of the VP1 gene involved in the 2016 epidemic in the Netherlands (*4*); the second closest subclade was B1, which includes EV-D68 strains from respiratory specimens of patients with AFM from the 2014 outbreak in North America (*11*).

## Conclusions

Since the large outbreak in 2014, EV-D68 infection has been recognized as a potential threat to patients with hematologic malignancies, especially HSCT recipients. Evidence that infections for these patients could be associated with severe respiratory disease is increasing (*4**,**12**).* As for other enteroviruses, infection with EV-D68 may be associated with neurologic features; and risk for severe illness is highest among children, elderly persons, and adults with underlying immune-compromising conditions. However, evidence addressing whether the EV-D68 infection is an incidental finding or a newly emerging cause of AFM is limited. The case reported here is 1 of only a handful of cases in which the EV-D68 genome was evidenced in CSF and not only in respiratory specimens, suggesting a possible causal association between EV-D68 and neurologic disease in adults. Recent findings in a mouse model provide evidence of a specific tropism of EV-D68 for spinal cord motor neurons, suggesting that direct viral injury, rather than a postinfection immune-mediated process, is the most likely mechanism of neuronal cell loss and paralysis (*13*). The phylogenetic analysis indicates that the strain detected in the patient described here, like one recently detected in a child with neurologic illness (*14*), is genetically linked to those involved in the recent outbreak in the Netherlands (*4*).

Immunocompromised adults, as well as children, may be at risk for neurologic complications from EV-D68 infection (*15*). This consideration, adding to the upsurge of recognized respiratory EV-D68 infections (*4*) and the scarcity of respiratory virus screening tests able to detect enteroviruses, highlights the value of including EV-D68 in the differential diagnosis for respiratory and neurologic complications in immunosuppressed patients (*12*).
