# Correction: Vol. 23, No. 10

**DOI:** 10.3201/eid2402.C12402

**Published:** 2018-02

**Keywords:** errata, erratum, correction

The author list has been corrected for Enterovirus D68–Associated Acute Flaccid Myelitis in Immunocompromised Woman, Italy (E. Giombini et al.). The article has been corrected online (https://wwwnc.cdc.gov/eid/article/23/10/17-0792_article).

